# The Neuroprotective Effects of Ginsenoside Rd Pretreatment in a Rat Model of Spinal Cord Ischemia-Reperfusion Injury

**DOI:** 10.21470/1678-9741-2021-0548

**Published:** 2023

**Authors:** Dong Jung Kim, Sunghee Han, Cheong Lim

**Affiliations:** 1 Department of Thoracic and Cardiovascular Surgery, Seoul National University College of Medicine, Seoul National University Bundang Hospital, Gyeonggi-do, South Korea.; 2 Department of Anesthesiology and Pain Medicine, Seoul National University College of Medicine, Seoul National University Bundang Hospital, Gyeonggi-do, South Korea.

**Keywords:** Ginsenosides, Spinal Cord, Paraplegia, Neuroprotection, Reperfusion Injury, Spinal Cord Ischemia, Superoxide Dismutase, Motor Neurons

## Abstract

**Introduction:**

Paraplegia may develop as a result of spinal cord ischemia-reperfusion injury in patients who underwent thoracoabdominal aortic surgery. The objective of this research is to determine the neuroprotective effects of ginsenoside Rd pretreatment in a rat model of spinal cord ischemia-reperfusion injury.

**Methods:**

Sprague-Dawley rats (n=36) were randomly assigned to three groups. The sham (n=12) and control (n=12) groups received normal saline orally. The Rd group (n=12) received ginsenoside Rd (100 mg/kg) orally 48 hours before the induction of spinal cord ischemia. Spinal cord ischemia was induced by aortic occlusion using a Fogarty balloon catheter in the Rd and control groups. A neurological assessment according to the motor deficit index and a histological evaluation of the spinal cord were performed. To evaluate the antioxidant activity of ginsenoside Rd, malondialdehyde levels and superoxide dismutase activity were determined. Further, the tissue levels of tumor necrosis factor-alpha and interleukin-1 beta were measured.

**Results:**

The Rd group showed significantly lower motor deficit index scores than did the control group throughout the entire experimental period (*P*<0.001). The Rd group demonstrated significantly greater numbers of normal motor neurons than did the control group (*P*=0.039). The Rd group exhibited decreased malondialdehyde levels (*P*<0.001) and increased superoxide dismutase activity (*P*=0.029) compared to the control group. Tumor necrosis factor-alpha and interleukin-1 beta tissue levels were significantly decreased in the Rd group (*P*<0.001).

**Conclusion:**

Ginsenoside Rd pretreatment may be a promising treatment to prevent ischemia-reperfusion injury in patients who undergo thoracoabdominal aortic surgery.

## INTRODUCTION

Spinal cord ischemia can develop in patients who undergo thoracoabdominal aortic surgery, potentially leading to subsequent paraplegia, which is a serious postoperative complication^[1]^. Several methods are used for preventing paraplegia, including cerebrospinal fluid drainage, deep hypothermia, and ischemic preconditioning. However, these approaches do not always provide sufficient protection^[2,3]^. Ischemia-reperfusion injury of the spinal cord, which is secondary to aortic cross-clamping and subsequent de-clamping during surgery, has been considered to contribute to the development of paraplegia after thoracoabdominal aortic surgery. Ischemia-reperfusion injury is closely related to increased oxidative stress, which is induced by overproduction of reactive oxygen species (ROS) or insufficient activity of antioxidants including superoxide dismutase (SOD)^[4,5]^. The overproduction of ROS and subsequent inflammatory cascade induced by ROS can cause neuronal cell damage and contribute to the development of ischemiareperfusion injury^[6]^. Infammatory cells produce large quantities of cytokines such as tumor necrosis factor-alpha (TNF-α) and interleukin-1 beta (IL-1β)^[7]^, which can exacerbate neuronal cell damage by stimulating other inflammatory cells and producing excessive free oxygen radicals^[8]^. Ginsenoside Rd is an active component of Ginseng root extract with multifunctional activity, including antioxidant, anti-inflammatory, and neuroprotective effects^[9,10,11]^. A previous study reported that ginsenoside Rd might have therapeutic effects on ischemia-reperfusion injury of the spinal cord^[11]^, but the neuroprotective effects of pretreatment with ginsenoside Rd remain undefined. This study aimed to determine the neuroprotective effects of ginsenoside Rd pretreatment in a rat model of spinal cord ischemia-reperfusion injury.

## METHODS

### Experimental Groups

All experimental procedures throughout the entire duration of the experiment were conducted according to the Guide for the Care and Use of Laboratory Animals. All procedures were reviewed and accepted by the institutional Animal Care and Use Committee. Male Sprague-Dawley rats (n=36), weighing between 300 and 350 g, were randomly assigned into the sham (n=12), control (n=12), and Rd (n=12) groups. The Rd group received ginsenoside Rd (100 mg/kg) orally, mixed with normal saline (1 mL), 48 hours before the induction of spinal cord ischemia. The dosage of ginsenoside Rd was determined according to previous studies^[11,12]^. In the sham and control groups, the same amount of normal saline was administered orally.

### Surgery

Anesthesia with 5% v/v isofurane was performed in all rats. For the maintenance of anesthesia after induction, 1.0-2.5% v/v isofurane was delivered via a face mask. After placing rats in the supine position, the fur around the neck and the left thigh was shaved. The tail artery was isolated and catheterized for monitoring distal arterial blood pressure and injection of heparin. To induce ischemia of the spinal cord, the left femoral artery was isolated and cannulated with a 2 Fr Fogarty catheter (Fogarty Arterial Embolectomy Catheter, Edwards Lifesciences, Irvine, California, United States of America). The end of the Fogarty catheter was located just distal to the left subclavian artery, approximately 11 cm from the cannulation site. To monitor the proximal arterial pressure, the left carotid artery was isolated and catheterized using a 20-gauge polyethylene (PE) catheter (BD Insyte, Becton Dickinson, Sandy, Utah, United States of America). To regulate the proximal arterial blood pressure up to 80 mmHg during the occlusion of the aorta, the PE catheter was connected to a reservoir filled with normal saline. To monitor body temperature, a rectal probe was used. During the aortic occlusion, the body temperature was kept constant at 37-38 °C using a heat lamp and a warming pad.

Heparin (150 U) was administered via the tail artery catheter after cannulation. Occlusion of the aorta was performed by infating the Fogarty catheter balloon with 0.05 mL of saline. Blood from the left carotid artery was concurrently collected into the blood reservoir to enable the regulation of proximal arterial blood pressure up to 80 mmHg during the occlusion of the aorta. Successful aortic occlusion was established by prompt loss of pulse and a reduction in distal arterial blood pressure. The Fogarty catheter balloon was defated after 10.5 min, and reperfusion of drained blood was performed, followed by the removal of all catheters and closure of the incisions. The rats were then sent back to their cages and allowed to recover from the anesthesia. Investigators blinded to the group allocation performed the surgical procedures. Although the same surgical techniques were used in all groups, ischemia of the spinal cord was only induced in the Rd and the control group.

### Neurologic Evaluation

The motor deficit index (MDI) score^[13]^ was recorded for the neurologic assessment by investigators blinded to the group allocation. The assessment started four hours after the rats had recovered from the anesthesia, and then repeated every 24 hours until two days after reperfusion. The MDI score was defined as the total score from ambulation and the placing/stepping reflex. Ambulation was graded from 0 to 4, by assessing the rats’ use of their lower extremities during walking, as follows: 0 = normal (symmetric and coordinated ambulation); 1 = toes fat under the body when walking but ataxia present; 2 = knuckle walking; 3 = movement in lower extremities but unable to knuckle walk; 4 = no movement or dragging of lower extremities. The placing/stepping reflex was graded from 0 to 2, by assessing the responses provoked by touching the rat’s hind paw. The reflex was graded as follows: 0 = normal (coordinated lifting and placing of the leg); 1 = weak; 2 = no stepping. After the neurologic evaluation, the total score was calculated as the MDI score for each rat. The maximum motor deficit was defined as an MDI score of 6.

### Histopathology

After assessment of their hindlimb motor function, the rats were anesthetized with isofurane, and 100 mL of heparinized saline was injected transcardially, followed by the removal and fixation of the lumbar spinal cord. After fixation, the spinal cord segments at the L3-L5 levels, which have been frequently used in previous studies for the characterization of histologic findings in this rat model^[14,15]^, were isolated and embedded in paraffin. Serial transverse sections (4 µm thick) were prepared and stained with hematoxylin and eosin. Three representative slides were selected from serial sections with intervals > 100 µm for the histologic examination in each rat. To evaluate the level of neuronal cell damage, the number of normal motor neurons was quantified by averaging the numbers of the three selected sections. The histologic features used to diferentiate normal and abnormal motor neurons were round nuclei, clearly visible nucleoli, and abundant cytoplasmic substances. Injured motor neurons were identified by darkly pyknotic nuclei, a pronounced eosinophilic cytoplasm, shrunken cellular bodies, and pericellular edema. The histologic examination was performed by investigators blinded to the group allocation, at a ×200 magnification.

### Malondialdehyde (MDA) Assay

Oxidative stress was evaluated by determining the levels of the lipid peroxidation end-product MDA. MDA levels were determined as per the manufacturer’s protocol (Abcam, Cambridge, United Kingdom, ab118970). In brief, homogenization of the spinal cord tissues was performed in phosphate-buffered saline on ice. Three cycles of freezing and defrosting of the homogenate in liquid nitrogen were conducted. After centrifugation, the supernatant was collected to measure MDA levels. The free MDA generated an MDA-thiobarbituric acid (TBA) adduct by reacting with TBA. The MDA-TBA adduct was quantified colorimetrically (optical density = 532 nm). To estimate the amount of MDA equivalents in the sample, an interpolation from the standard curve was performed. MDA levels were expressed as nmol/mg of tissue protein.

### Superoxide Dismutase Assay

SOD activity was quantified using an SOD assay kit (Catalogue No. 706002; Cayman Chemical Company, Ann Arbor, Michigan, United States of America). For the measurement of SOD activity, hypoxanthine and xanthine oxidase were used as a superoxide generator, and nitrobluetetrazolium served as a superoxide indicator. Preparation of all reagents and specimens was performed as per the manufacturer’s protocol. Briefly, spinal cord tissue homogenates were prepared in the same way as previously described for the MDA assay. After centrifugation of homogenates, the supernatant was collected to measure SOD activity. To analyze the changes in absorbance, a plate reader (BioTek Instruments, Winooski, Vermont, United States of America) was used at 450 nm. SOD activity was expressed as U/mg protein.

### Enzyme-Linked Immunosorbent Assay (ELISA)

Infammatory markers were evaluated using an ELISA technique. Homogenization of the spinal cord tissues was performed on ice with radioimmunoprecipitation assay bufer. After the centrifugation of the homogenates, the levels of TNF-α and IL-1β in the supernatant were measured using a commercially available ELISA kit (AB100785 and AB100768; Abcam, Cambridge, United Kingdom). The assays were used according to the manufacturer’s protocol. Briefly, antibodies specific to TNF-α and IL-1β were used to coat the microplate kit. The absorbance was spectrophotometrically measured at 450 nm (SpectraMax M2, Molecular Devices, Sunnyvale, California, United States of America). The results were expressed as picograms per milligram (pg/mg) protein.

### Statistical Analysis

IBM Corp. Released 2017, IBM SPSS Statistics for Windows, version 25.0, Armonk, NY: IBM Corp. was used for statistical analyses. All data are expressed as mean ± standard error of mean. Hindlimb motor function was analyzed using repeated-measures analysis of variance (ANOVA) followed by Dunnett’s post hoc test and the Mann-Whitney U test with Bonferroni correction. Statistical analyses of other data were performed using one-way ANOVA followed by Tukey’s honestly significant diference post hoc test. *P*-values < 0.05 were considered statistically significant.

## RESULTS

### Hindlimb Motor Function

**Fig. 1:**
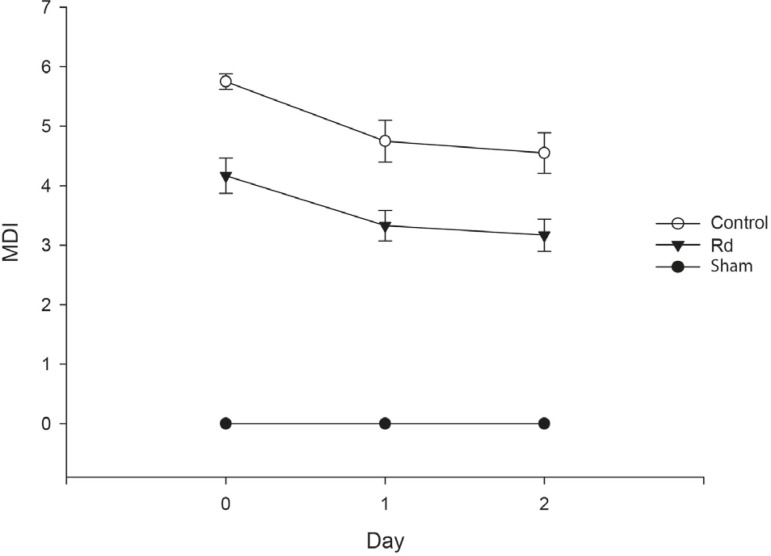
Motor deficit index (MDI) evaluated at days 0, 1, and 2 after induction of spinal cord ischemia-reperfusion injury. Each symbol represents the MDI score of each group. Rats in the sham group demonstrated normal motor function as represented by an MDI score of 0. MDI scores were significantly lower in the Rd group than in the control group throughout the entire experimental period. All values are presented as mean ± standard error of mean.

To assess the neuroprotective effects of pretreatment with ginsenoside Rd in rats, hindlimb motor function was evaluated daily using MDI scores. All rats except one in the control group survived until the last neurological evaluation was performed on day two. The MDI scores for each group are shown in Figure 1. In the sham group, all rats demonstrated normal motor function as represented by MDI scores of 0 throughout the entire experimental period. The changes in MDI scores over time were significantly diferent between groups (repeated-measures ANOVA, *P*=0.002). A significant diference in MDI scores was found between the Rd and control group (Dunnett’s post hoc test, *P*<0.001). On day zero, the Rd group exhibited significantly lower MDI scores than did the control group (4.17±0.30 *vs.* 5.75±0.13, *P*<0.001). This trend continued until 48 hours after reperfusion (day one: 3.33±0.26 *vs.* 4.75±0.35, *P*=0.008; day two: 3.17±0.27 *vs.* 4.55±0.34, *P*=0.011).

### Neuronal Survival

**Fig. 2:**
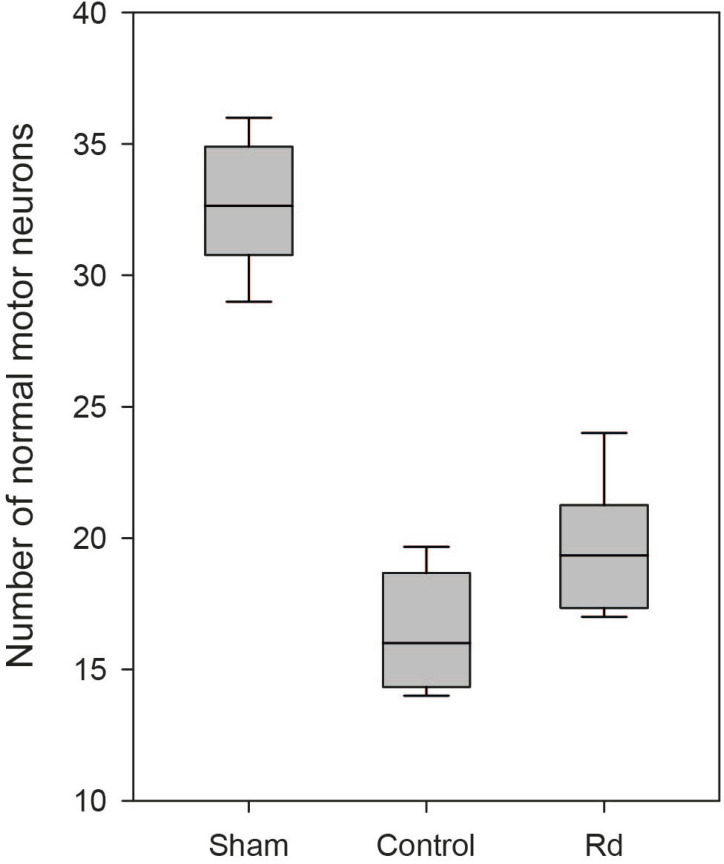
Boxplot of the number of normal motor neurons in each group. Significantly fewer normal motor neurons were detected in groups with spinal cord ischemia than in the sham group (P<0.001). Significantly more normal motor neurons were observed in the Rd group compared to the control group (P=0.039).

**Fig. 3:**
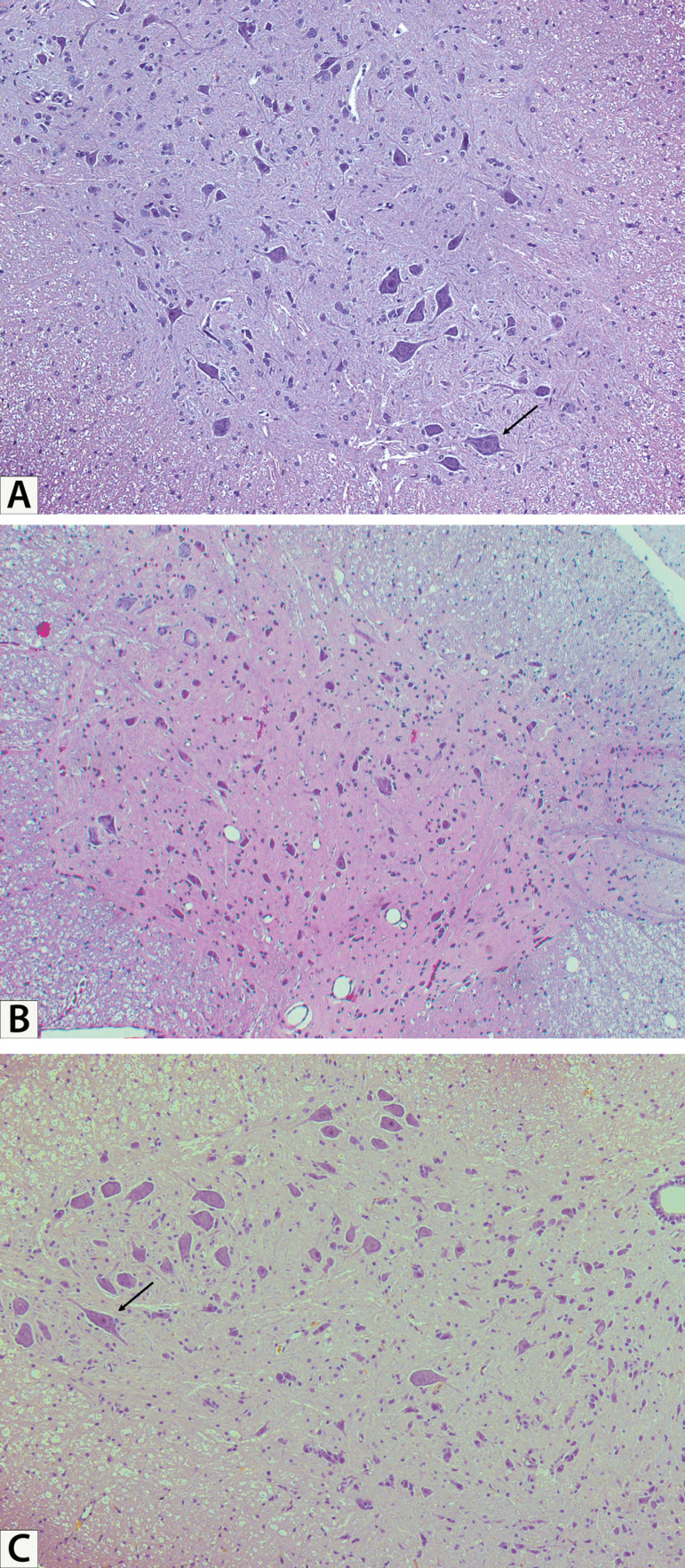
Representative photomicrographs of spinal cord sections in each group (stained with hematoxylin and eosin, ×200 magnification). A) The sham group, demonstrating motor neurons with normal histologic features (arrow). B) The control group, indicating injured motor neurons with marked vacuolization and shrunken cellular bodies. No motor neuron with normal histologic features is depicted. C) The Rd group, exhibiting relatively well-preserved normal motor neurons (arrow).

To evaluate the neuroprotective effects of ginsenoside Rd on neuronal survival in rats, a histologic examination of spinal cord tissues was performed as previously described. While motor neurons with normal histologic features were depicted in the control group, the Rd group also exhibited relatively well-preserved normal motor neurons. Representative photomicrographs for each group are shown in Figures 3A, 3B, and 3C. The number of normal motor neurons in the Rd group was significantly greater than that in the control group (19.58±0.87 *vs.* 16.34±0.82, *P*=0.039) and significantly lower than that in the sham group, (19.58±0.87 *vs.* 32.63±0.85; *P*<0.001). The number of normal motor neurons in each group is shown in Figure 2.

### Oxidative Stress

To investigate putative neuroprotective mechanisms, we assessed the antioxidative effects of ginsenoside Rd on oxidative stress in rat spinal cord tissue. MDA levels and SOD activity in spinal cord tissue were measured 48 hours after the induction of ischemia-reperfusion injury. The control group exhibited significantly higher MDA levels than did the sham group (3.736±0.165 *vs.* 3.038±0.013 nmol/mg, *P*<0.001; Figure 4A), while the Rd group demonstrated significantly lower MDA levels than did the control group (3.021±0.018 *vs.* 3.736±0.165, nmol/mg, *P*<0.001; Figure 4A). SOD activity was significantly higher in the Rd group than in the control group (66.495±1.092 *vs.* 57.255±2.023, U/mg, *P*=0.029; Figure 4B). There were no significant differences in MDA levels and SOD activity between the Rd and sham groups (*P*>0.05; Figures 4A & 4B).

**Fig. 4A:**
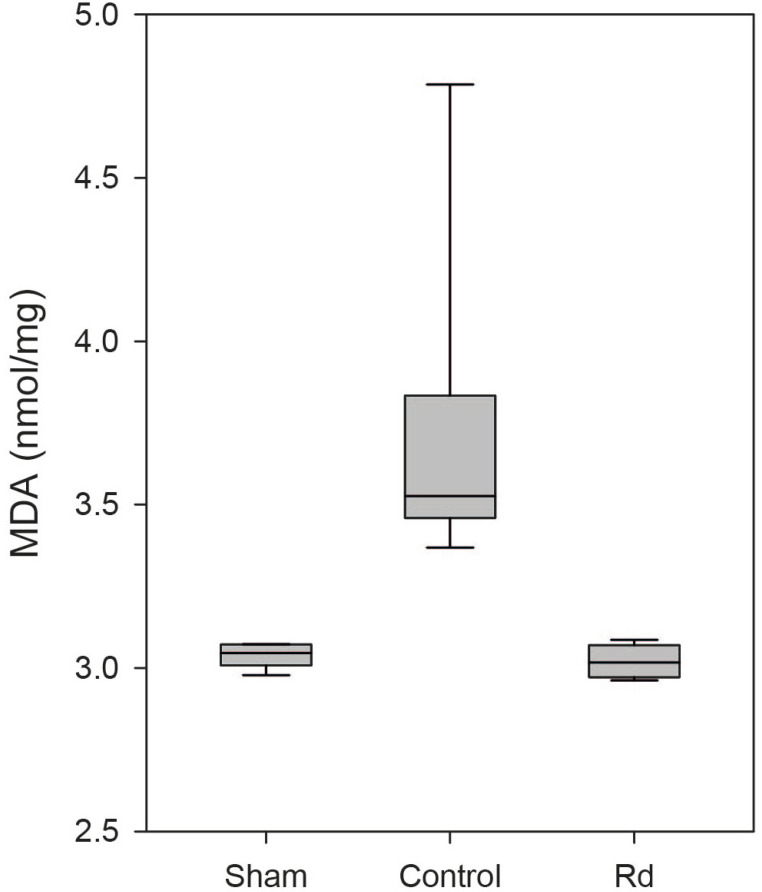
Boxplot of malondialdehyde (MDA) levels in each group. Significantly higher MDA levels were observed in the control group compared to the sham group (P<0.001). Significantly lower MDA levels were observed in the Rd group compared to the control group (P<0.001). No significant diference was observed between the Rd and sham groups (P>0.05).

**Fig. 4B:**
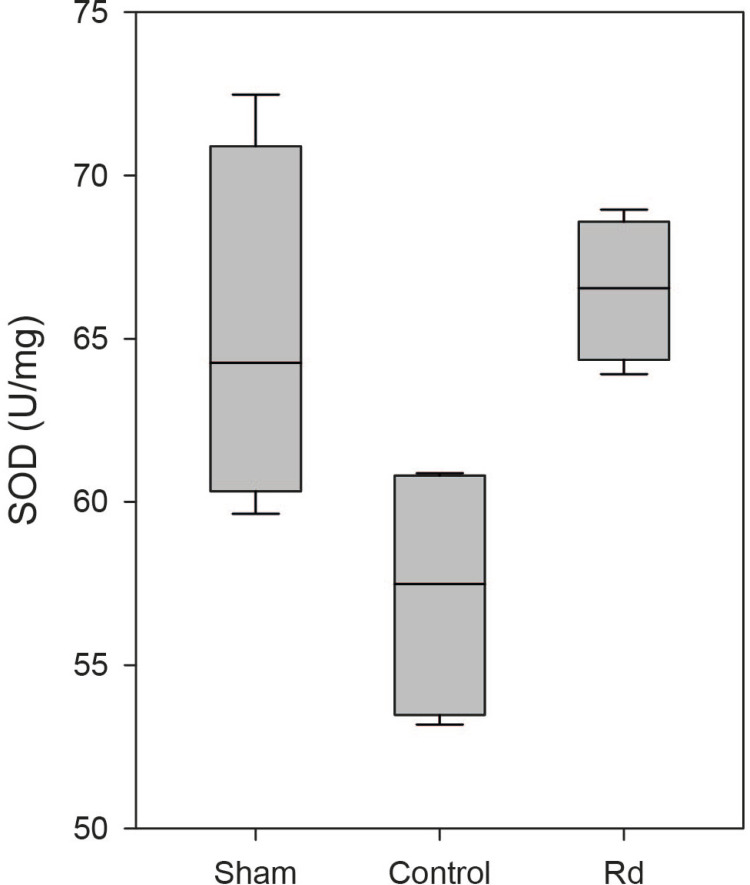
Boxplot of superoxide dismutase (SOD) activity in each group. SOD activity was significantly higher in the Rd group than in the control group (P=0.029). There was no significant diference in SOD activity between the sham and Rd groups (P>0.05).

### Infammatory Responses

To assess the anti-inflammatory effects of ginsenoside Rd, the levels of key pro-inflammatory cytokines, TNF-α, and IL-1β in spinal cord tissue were evaluated 48 hours after the induction of ischemia-reperfusion injury. The control group exhibited significantly higher levels of TNF-α and IL-1β than did the sham group (*P*<0.001; Figures 4C and 4D). The Rd group demonstrated significantly lower levels of TNF-α than did the control group (6.04±0.15 *vs.* 16.84±1.46, pg/mg protein, *P*<0.001; Figure 4C). No significant diference in TNF-α levels was found between the Rd and sham groups (*P*>0.05; Figure 4C). IL-1β levels in the Rd group were significantly lower than those in the control group (23.78±0.77 *vs.* 39.62±1.19, pg/mg protein, *P*<0.001; Figure 4D) and significantly higher than those in the sham group (23.78±0.77 *vs.* 17.91±0.80, pg/mg protein, *P*=0.002; Figure 4D).

**Fig. 4C:**
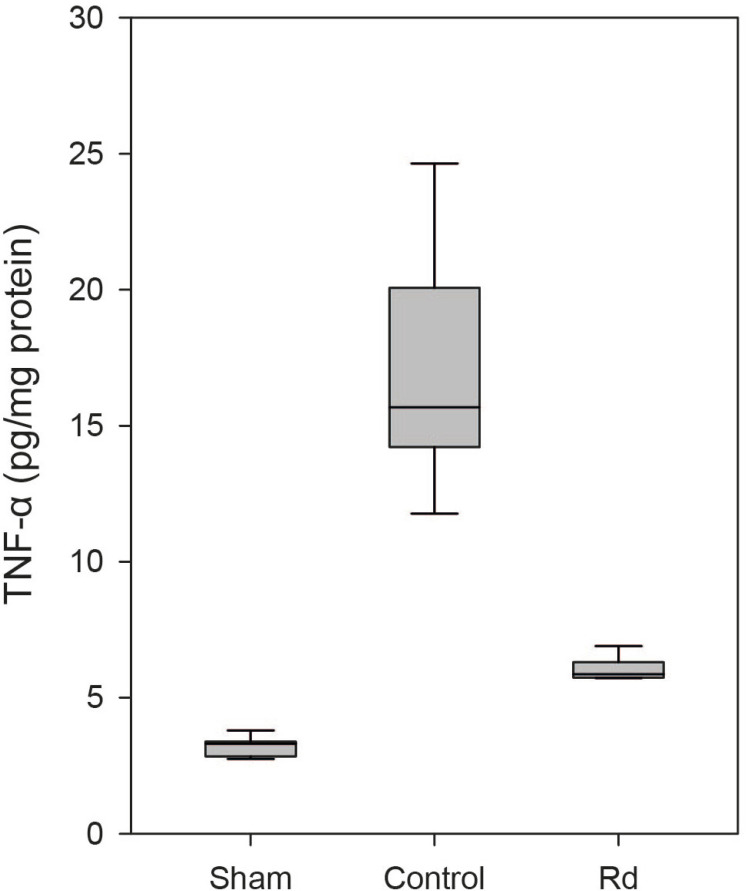
Boxplot of the levels of tumor necrosis factor-alpha (TNF-α) in each group. The level of TNF-α was significantly lower in the Rd group than in the control group (P<0.001). There was no significant diference in the levels of TNF-α between the Rd and sham groups (P>0.05).

**Fig. 4D:**
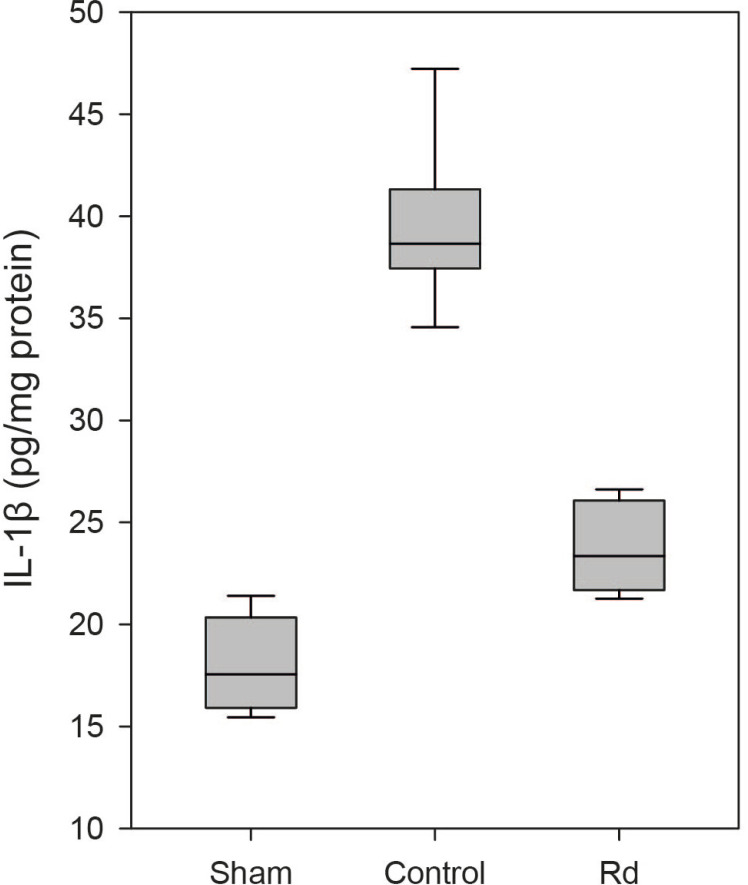
Boxplot of the levels of interleukin-1 beta (IL-1β) in each group. Significantly lower levels of IL-1β were observed in the Rd group compared to those in the control group (P<0.001). Conversely, significantly higher levels of IL-1β were observed in the Rd group compared to those in the sham group (P=0.002).

## DISCUSSION

Paraplegia is an important postoperative complication of thoracoabdominal aortic surgery^[1]^. Previous studies have reported an incidence as high as 23%, but the adoption of methods to prevent paraplegia has reduced the incidence to 4-7%^[16]^. Nevertheless, it is still considered a serious complication that requires more efficient preventative methods. It develops due to temporary ischemia of the spinal cord as a result of the transient loss of blood flow during the operation. Ischemia-reperfusion injury may occur when the bloodstream to the spinal cord is resumed after temporary ischemia. Infammatory responses and ROS underpin the etiology of this injury^[17,18]^. ROS are implicated in the stimulation of inflammatory cells. Neutrophils stimulate monocytes to generate a diversity of cytokines in ischemic tissue^[19,20]^. This study demonstrates that pretreatment with ginsenoside Rd promotes neurological recovery and maintains the number of normal motor neurons in a rat model of spinal cord ischemia-reperfusion injury. We demonstrate that ginsenoside Rd may protect tissues from oxidative stress by decreasing MDA levels and increasing SOD activity. Furthermore, we show that ginsenoside Rd may have anti-inflammatory effects, indicated by lower levels of pro-inflammatory cytokines such as TNF-α and IL-1β in the ginsenoside Rd pretreatment group.

Ginseng is a broadly used traditional medicine for the treatment of various diseases, particularly in Northeastern Asia^[21]^. Ginsenoside Rd is a major active component in ginseng extract. It is fat-soluble and can cross the blood-brain barrier^[22]^. Ginsenoside Rd has multipotent effects including antioxidant, anti-inflammatory, and neuroprotective properties^[9,10,11]^. Previous studies have indicated that ginsenoside Rd may have neuroprotective effects in various diseases, including cerebral ischemia^[23]^, neuronal injury^[12]^, and spinal cord ischemia-reperfusion injury^[11]^. Several studies using a transient middle cerebral artery occlusion rat model have pointed to the beneficial effects of pretreatment with ginsenoside Rd on cerebral ischemia^[23]^. Several *in vitro* and *in vivo* studies have demonstrated that ginsenoside Rd pretreatment before ischemic stroke may decrease infarct volume, promote neuronal survival, and improve neurological function^[23,24]^. A previous investigation demonstrated that ginsenoside Rd significantly enhanced the motor function of rats by debilitating spinal cord tissue injury and promoting neuronal survival in a rat model of spinal cord injury^[12]^. Another study demonstrated that ginsenoside Rd may exert therapeutic effects on spinal cord ischemia-reperfusion injury by suppression of apoptosis^[11]^. In our study, pretreatment with ginsenoside Rd (100 mg/kg) 48 hours before spinal cord ischemia-reperfusion injury effectively enhanced the recovery of neurological function. In line with the behavioral test results, more normal motor neurons were preserved in rats in the Rd group compared to the control group.

Ischemia-reperfusion injury is strongly associated with increased oxidative stress^[4]^. Oxidative stress is induced by a disproportion between the overproduction of ROS and inadequate activity of endogenous antioxidative systems^[5]^. Although ROS are normally produced during cellular metabolism, the excessive generation of ROS can be cytotoxic, because of their ability to react with and damage essential cellular structural elements, resulting in cellular dysfunction and cell death^[25]^. Excessive amounts of ROS also react with polyunsaturated fatty acids and induce intracellular lipid peroxidation, resulting in the generation of high levels of lipid peroxides and hydroperoxides such as MDA. These molecules inhibit enzyme systems bound to cellular membranes, and this result in the disruption of cellular integrity^[17]^. Antioxidants such as SOD are one of the essential mechanisms underlying the defense against ROS-induced cellular damage^[26]^. However, antioxidant activity may be attenuated by cellular damage when oxidative stress is increased. A previous investigation demonstrated that ginsenoside Rd can protect neural tissues against oxidative stress by decreasing MDA levels and increasing SOD activity^[24]^. The antioxidative activity of ginsenoside Rd may be due to its chemical structure, which is similar to that of steroids. Liu et al.^[27]^ have suggested that its steroid-like structure enables it to access intracellular locations and may thus contribute to its properties as an antioxidant. In our study, ischemia-reperfusion injury significantly induced MDA production in the control group. Consistent with previous studies, our study shows that Rd pretreatment attenuates oxidative stress by reducing MDA production and enhancing SOD activity in spinal cord tissues.

Inflammation plays a significant role during the acute phase of ischemia-reperfusion injury^[28]^. Although ROS directly damage cell membranes, they can also trigger neutrophil infiltration to the site of injury. Activated neutrophils contribute to endothelial damage by producing inflammatory mediators, including free oxygen radicals^[29]^. Accumulated neutrophils mediate the inflammatory cascade by releasing pro-inflammatory cytokines, such as TNF-α and IL-1β, leading to spinal cord ischemic injury. TNF-α contribute to endothelial damage by promoting the production of endothelial leukocyte adhesion molecules^[30]^. Moreover, TNF-α has neurotoxic effects and promotes apoptosis in neurons^[29]^. IL-1β is a pro-inflammatory cytokine that plays a critical role in leukocyte infiltration and neuronal apoptosis^[31]^. Both TNF-α and IL-1β contribute to the generation of interleukin-6, which facilitates the proliferation of B cells and results in the accumulation of neutrophils and the overproduction of ROS^[19]^. These cytokines may stimulate other inflammatory cells and play an important role in increasing the extent of ischemia-reperfusion injury by the production of ROS, which can exacerbate neuronal damage^[8]^. Therefore, assaying these cytokines in spinal cord tissue may be useful for quantifying the extent of ischemia-reperfusion injury^[32]^. In the current study, the Rd group demonstrated significantly lower cytokine levels than did the control group.

### Limitations

This study has several limitations. For the application of these results in clinical practice, larger samples of animals in each group should be included. Further, the optimal dosing and timing of the administration of ginsenoside Rd should be studied in a dose-dependent manner.

## CONCLUSION

Pretreatment with ginsenoside Rd significantly promoted neurological recovery and preserved a greater number of normal motor neurons in a rat model of spinal cord ischemia-reperfusion injury. We demonstrate that these effects may be underpinned by attenuated oxidative stress and decreased inflammatory responses. To the best of our knowledge, this is the first study to demonstrate the neuroprotective effects of ginsenoside Rd pretreatment in a rat model of spinal cord ischemia-reperfusion injury by evaluating antioxidative effects, pro-inflammatory cytokine levels, and functional outcomes. Although further investigations are required, the potential neuroprotective effects of ginsenoside Rd pretreatment ofer a promising strategy for the prevention of ischemia-reperfusion injury in patients who undergo thoracoabdominal aortic surgery.

## References

[1] Wan IY, Angelini GD, Bryan AJ, Ryder I, Underwood MJ (2001). Prevention of spinal cord ischaemia during descending thoracic
and thoracoabdominal aortic surgery. Eur J Cardiothorac Surg.

[2] Zvara DA, Colonna DM, Deal DD, Vernon JC, Gowda M, Lundell JC (1999). Ischemic preconditioning reduces neurologic injury in a rat model
of spinal cord ischemia. Ann Thorac Surg.

[3] Salzano RP Jr, Ellison LH, Altonji PF, Richter J, Deckers PJ (1994). Regional deep hypothermia of the spinal cord protects against
ischemic injury during thoracic aortic cross-clamping. Ann Thorac Surg.

[4] Torres S, Salgado-Ceballos H, Torres JL, Orozco-Suarez S, Díaz-Ruíz A, Martínez A (2010). Early metabolic reactivation versus antioxidant therapy after a
traumatic spinal cord injury in adult rats. Neuropathology.

[5] Sullivan PG, Krishnamurthy S, Patel S P, Pandya JD, Rabchevsky AG (2007). Temporal characterization of mitochondrial bioenergetics after
spinal cord injury. J Neurotrauma.

[6] Akira S, Hirano T, Taga T, Kishimoto T (1990). Biology of multifunctional cytokines: IL 6 and related molecules
(IL 1 and TNF). FASEB J.

[7] David S, Zarruk JG, Ghasemlou N (2012). Infammatory pathways in spinal cord injury. Int Rev Neurobiol.

[8] Dawson TM, Dawson VL, Snyder SH (1994). Molecular mechanisms of nitric oxide actions in the
brain. Ann N Y Acad Sci.

[9] Guan YY, Zhou JG, Zhang Z, Wang GL, Cai BX, Hong L (2006). Ginsenoside-Rd from panax notoginseng blocks Ca2+ infux through
receptor- and store-operated Ca2+ channels in vascular smooth muscle
cells. Eur J Pharmacol.

[10] Zhang YX, Wang L, Xiao EL, Li SJ, Chen JJ, Gao B (2013). Ginsenoside-Rd exhibits anti-inflammatory activities through
elevation of antioxidant enzyme activities and inhibition of JNK and ERK
activation in vivo. Int Immunopharmacol.

[11] Wang B, Zhu Q, Man X, Guo L, Hao L (2014). Ginsenoside Rd inhibits apoptosis following spinal cord
ischemia/reperfusion injury. Neural Regen Res.

[12] Cong L, Chen W (2016). Neuroprotective effect of ginsenoside rd in spinal cord injury
rats. Basic Clin Pharmacol Toxicol.

[13] Taira Y, Marsala M (1996). Effect of proximal arterial perfusion pressure on function,
spinal cord blood flow, and histopathologic changes after increasing
intervals of aortic occlusion in the rat. Stroke.

[14] Saito T, Saito S, Yamamoto H, Tsuchida M (2013). Neuroprotection following mild hypothermia after spinal cord
ischemia in rats. J Vasc Surg.

[15] Kim J, Hwang J, Huh J, Nahm SF, Lim C, Park S (2012). Acute normovolemic hemodilution can aggravate neurological injury
after spinal cord ischemia in rats. Anesth Analg.

[16] George R (2015). Spinal cord ischemia after thoracoabdominal aortic
procedures. Heart India.

[17] Boyle EM Jr, Pohlman TH, Cornejo CJ, Verrier ED (1996). Endothelial cell injury in cardiovascular surgery:
ischemia-reperfusion. Ann Thorac Surg.

[18] Jaeschke H (1996). Preservation injury: mechanisms, prevention and
consequences. J Hepatol.

[19] McMillen MA, Huribal M, Sumpio B (1993). Common pathway of endothelial-leukocyte interaction in shock,
ischemia, and reperfusion. Am J Surg.

[20] Schwartz MD, Repine JE, Abraham E (1995). Xanthine oxidase-derived oxygen radicals increase lung cytokine
expression in mice subjected to hemorrhagic shock. Am J Respir Cell Mol Biol.

[21] Lü JM, Yao Q, Chen C (2009). Ginseng compounds: an update on their molecular mechanisms and
medical applications. Curr Vasc Pharmacol.

[22] Yang L, Deng Y, Xu S, Zeng X (2007). In vivo pharmacokinetic and metabolism studies of ginsenoside
Rd. J Chromatogr B Analyt Technol Biomed Life Sci.

[23] Ye R, Kong X, Yang Q, Zhang Y, Han J, Zhao G (2011). Ginsenoside Rd attenuates redox imbalance and improves stroke
outcome after focal cerebral ischemia in aged mice. Neuropharmacology.

[24] Ye R, Yang Q, Kong X, Han J, Zhang X, Zhang Y (2011). Ginsenoside Rd attenuates early oxidative damage and sequential
inflammatory response after transient focal ischemia in rats. Neurochem Int.

[25] Beattie MS, Hermann GE, Rogers RC, Bresnahan JC (2002). Cell death in models of spinal cord injury. Prog Brain Res.

[26] Vaziri ND, Lee YS, Lin CY, Lin VW, Sindhu RK (2004). NAD(P)H oxidase, superoxide dismutase, catalase, glutathione
peroxidase and nitric oxide synthase expression in subacute spinal cord
injury. Brain Res.

[27] Liu ZQ, Luo XY, Liu GZ, Chen Y P, Wang ZC, Sun YX (2003). In vitro study of the relationship between the structure of
ginsenoside and its antioxidative or prooxidative activity in free radical
induced hemolysis of human erythrocytes. J Agric Food Chem.

[28] Tian DS, Xie MJ, Yu ZY, Zhang Q, Wang YH, Chen B (2007). Cell cycle inhibition attenuates microglia induced inflammatory
response and alleviates neuronal cell death after spinal cord injury in
rats. Brain Res.

[29] Wang CX, Nuttin B, Heremans H, Dom R, Gybels J (1996). Production of tumor necrosis factor in spinal cord following
traumatic injury in rats. J Neuroimmunol.

[30] Klebanof SJ, Vadas MA, Harlan JM, Sparks LH, Gamble JR, Agosti JM (1986). Stimulation of neutrophils by tumor necrosis
factor. J Immunol.

[31] Aloisi F, Borsellino G, Caré A, Testa U, Gallo P, Russo G (1995). Cytokine regulation of astrocyte function: in-vitro studies using
cells from the human brain. Int J Dev Neurosci.

[32] Miranda KM, Espey MG, Wink DA (2001). A rapid, simple spectrophotometric method for simultaneous
detection of nitrate and nitrite. Nitric Oxide.

